# Investigations into the Terminations of the Cutaneous Nerves in Man

**Published:** 1869-10

**Authors:** 

**Affiliations:** Liége


Article II.—*Investigations into the Terminations of the Cutaneous Nerves in Man.* By Dr. Grandry, Liége. (Robins' Journal de la Physiol et de l'Anatom.)

In this article the mode of termination of the nerves in the corpuscles of Meissner will alone be discussed. As to the modes of termination indicated by Langerhaus, and to that in the tactili papillæ indicated by Odenius, they shall be discussed in a subsequent article.

The history of the corpuscles of Meissner has been discussed for a number of years, and the different observers by no means agree concerning the intimate structure of this terminal nervous organ, and especially about the mode of termination of the fibre itself. The bibliographical question having been very well treated in the paper of M. Rouget,* it is not necessary to return to it here. I shall only concern myself with the terminal extremity of the nerve, and with its relations to the envelope of the corpuscle.

Before describing the result of my observations, I shall give, in detail, the process followed. Fragments of skin of the fingers taken from an arm amputated in consequence of a white swelling of the elbow, were placed in different re-agents—acetic acid, bichromate of potass., chloride of gold, hyperosmic acid—and it was by the use of the re-agents that I obtained the results which I shall describe. With the solution of the bichromate of

* *Archives de Physiologie Normale et Pathologiques.* Publiées par M. M. Brown-Sequard, etc. No. 5, 1868.

potass., or the solution of Müller, I succeeded best in seeing the termination. For the course of the nerve itself, hyperosmic acid should be used in preference.

Several points should be considered in the examination of the corpuscles of Meissner: 1st, the envelope; 2nd, the central bulb; 3rd, the course of the fibre; 4th, its mode of termination.

The envelope is formed of dense connective tissue, as Kölliker admits, and I believe with this observer, that the transverse nuclei which are seen at the surface, especially under the action of acetic acid, belong to the connective tissue. In any case, I have not been able to discover the spiral arrangement of the fibre reduced to the condition of axis-cylinder. I believe the central bulb to be altogether analagous to that of the Pacinian corpuscles, and therefore I refer to what I have said of the reaction of chloride of gold upon these corpuscles. However, there is one fact to which I desire to direct attention successively. It is the existence, in the interior, of this central bulb of morphological elements, a fact denied by some observers.

The course of the medullary fibre is exactly that described by the majority of observers; that is to say, that it forms a spiral with large coils around the corpuscle, and thus reaches the upper portion of the corpuscle, still remaining within the envelope, without penetrating the central bulb. Does the medullary fibre always advance toward the exterior of the central bulb? I do not believe it, for I have distinctly seen, under osmic acid, the fibre in the interior of the envelope, and the solution of Müller has equally well demonstrated it to me penetrating into the central bulb still provided with medullary matter. Many opinions are held concerning its mode of termination. Rouget says that the fibre terminates in the central mass, being continuous with it, so that the central bulb may be considered as the fibre enlarged, and according to this author the morphological elements should be analagous to those found in the terminal plates of the muscles. I, however, have ascertained that the nerve does not terminate by becoming continuous with the central mass, but, indeed, with the morphological elements which are found in its interior; and this suggests thoroughly the terminal enlargements of the Pacinian corpuscles. Moreover, I have made the following observations upon transverse sections of the corpuscles of

Meissner, from the pulp of the fingers, treated by the solution of Müller.

I observed in their interior bodies more or less spherical, granular, varying (in diameter) from $0^{mm}008$ to $0^{mm}01$, which seemed frequently isolated and having no relation with a fibre or with the envelope, and situated most frequently close to the vertical axis of the corpuscle. Attentive examination, and good preparations, permit the detection, besides the spherical bodies, of excessively pale fibres lying in different planes not anastomosing, separating from the envelope, not losing themselves in the granular matter, but becoming continuous with the morphological granular elements, so that the latter appear pediculated. A fact well worth noticing is that the fibres separating from the envelope do not go directly to the granular bodies, but describe sinuosities more or less large before reaching them. I have seen a nerve fibre with double contour penetrating into the interior become reduced to the state of axis-cylinder and become continuous with the granular bodies.

At this point the question may be asked: Does the fibre terminate in a brush of more minute fibres reduced to the state of axis-cylinder; or, in describing spirals around the corpuscle, does it send out lateral branches which, passing on, terminate in the central bulb? I could not actually determine this. I believe, however, that there are lateral fibres sent out by the principal fibre; but I am not indisposed to believe that at its termination the medullary fibre subdivides into several branches. I have seen fibres separating from the lateral parts and terminating in the central mass by granular enlargements. What I have said concerning one fibre is applicable to corpuscles which receive several of them. Each one of them sends off branches which terminate by a swollen extremity in the central bulb.

If, now, comparison be made between the corpuscles of Pacini and the corpuscles of Meissner, it will appear that, in the corpuscles of Meissner, the termination of the nerve does not differ from that of the corpuscle of Pacini; that in both cases the fibre is terminated by a swollen extremity; the only difference being that in the corpuscles of the skin the fibre furnishes a greater number of terminal extremities, although three of them might have been found in the corpuscles of Pacini. I have not been able to determine whether the axis-cylinder behaves in the corpuscles of Meissner as in those of Pacini; that is to say, whether they divide into fibrillæ upon reaching their terminal enlargement.

My investigations have been conducted in the histological laboratory of the Faculty of Medicine of Paris, under the direction of M. Robin (Professor), to whom I here testify my gratitude for his wise counsels given to me upon all occasions. W. H.

